# Pharmaco-Toxicological Assessment of the Combined Cytotoxic Effects of Digoxin and Betulinic Acid in Melanoma Cells

**DOI:** 10.3390/life12111855

**Published:** 2022-11-11

**Authors:** Robert Rednic, Ioana Macasoi, Iulia Pinzaru, Cristina Adriana Dehelean, Mirela-Cleopatra Tomescu, Monica Susan, Horea Feier

**Affiliations:** 1 Faculty of Medicine, “Victor Babes” University of Medicine and Pharmacy Timisoara, Eftimie Murgu Square No. 2, 300041 Timisoara, Romania; 2 Faculty of Pharmacy, “Victor Babes” University of Medicine and Pharmacy Timisoara, Eftimie Murgu Square No. 2, 300041 Timisoara, Romania; 3 Research Center for Pharmaco-Toxicological Evaluations, Faculty of Pharmacy, “Victor Babes” University of Medicine and Pharmacy Timisoara, Eftimie Murgu Square No. 2, 300041 Timisoara, Romania

**Keywords:** betulinic acid, digoxin, F-actin fibers, HET-CAM assay, melanoma cells, synergic effect

## Abstract

Betulinic acid, a small molecule from pentacyclic triterpenes class, has been widely studied for its antitumor activity, revealing that it induces the apoptosis of tumor cells in a selective manner. In recent years, digoxin, a cardiac glycoside found particularly in the plant species *Digitalis lanata*, has drawn interest for its potential antitumor properties. The present study was designed to evaluate the antimelanoma potential of betulinic acid (BA), digoxin (DG), and their association (DG + BA). In vitro assessments were performed 24 h post-treatment on two human melanoma cell lines (SK-Mel-28 and RPMI-7951). In addition, the potential irritant effects of the test samples were evaluated using the chorioallantoic membrane of hen’s eggs. BA and DG exhibit a concentration-dependent cytotoxic activity, with the combination of the two having a more marked effect on the decrease in cell viability (~17% for SK-Mel-28 cells and ~23% for RPMI-7951 cells). Further, morphological changes (rounding of the cells and their separation from the plaque) and alterations in the nucleus and actin fibers (condensation of chromatin and actin fibers, formation of apoptotic bodies) were observed, indicating an apoptotic-like process. Moreover, no irritating effects were observed in ovo. As a result, DG + BA acid may have synergistic potential in the antitumor treatment of melanoma, but future studies are needed in order to clarify the biological mechanisms involved.

## 1. Introduction

There have been significant advances in the diagnosis and treatment of cancer, but it remains a major health issue on a global scale. Accordingly, over 19 million new cancer cases and over 10 million cancer-related deaths were reported in 2020 [1]. Globally, skin cancer is one of the most prevalent types of cancer, since both melanoma and non-melanoma skin cancer have reached epidemic proportions in recent years [2]. In 2020, there were approximately 350,000 cases of melanoma of the skin, representing 1.7% of all skin cancer cases diagnosed [3]. Even though melanoma only constitutes a small percentage of all skin cancers, it is responsible for more than 90% of all skin cancer-related deaths [4]. Aside from chemotherapy, radiotherapy, and photodynamic therapy, more modern therapeutic approaches such as immunotherapy and targeted therapy are available for treating melanoma. However, the current therapeutic options for melanoma pose problems due to toxic reactions as well as resistance of the cancer cells to treatment, although there have been significant advances in this area [5].

Plants, and particularly their secondary metabolites, are an important source of biologically active compounds [6,7]. Pentacyclic triterpenic acids have become a topic of intensive research in oncology therapy due to their wide range of biological actions [8]. Betulinic acid is one of these natural compounds, which in addition to having anti-inflammatory and anti-parasitic properties, has also been demonstrated to have antitumor properties. Mechanisms involved in the antitumor action are complex and incompletely understood [9]. In 1995, Pisha et al. conducted the first study to investigate betulinic acid’s antimelanoma activity [10]. In the years since then, betulinic acid has been intensively studied for its antitumor activity, revealing that it induces the apoptosis of tumor cells in a selective manner and, in addition, controls cytochrome C and Smac release at the mitochondrion level [11].

Digoxin is a cardiac glycoside found particularly in the plant species *Digitalis lanata*. It is considered one of the oldest drugs in medical practice and is currently used in cardiovascular diseases, especially in the treatment of heart failure and certain cardiac arrhythmias [12]. Digoxin exerts cardiac effects by inhibiting the Na+/K+ ATPase pump, leading to an increase in intracellular calcium concentration as well as an increased myocardial contractility [13]. In recent years, cardiotonic glycosides have attracted attention due to the possible antitumor properties, demonstrating their ability to inhibit tumor cell proliferation and induction of apoptosis [14]. It has also been highlighted that cardiac glycosides are cytotoxic to melanoma cells, whereas this effect is not evident in healthy human melanocytes. A possible explanation for this biological activity can be found in cardiac glycosides, such as digoxin, which inhibit the ATP1A1 Na+/K+ pump. This pump is crucial in maintaining ion gradients at the plasma membrane level, contributing to substrate transport between cells [15]. The use of cardiotonic glycosides alone did not have an important effect on the regression of xenografts from patients, but a synergistic effect was found when combined with inhibitors of the MAPK pathway [16].

In light of the fact that tumor cells employ various molecular mechanisms in order to survive, combined therapy may be an effective treatment option since it has the potential to simultaneously target most of these molecular mechanisms. As a consequence, combined therapy provides a major advantage by reducing resistance to the treatment [17]. As a result, associating several biologically active substances can usually achieve greater effectiveness because each substance can compensate for the disadvantages of the other [18]. There has been a particular focus on the synergistic effects of phytochemicals. Compounds derived from nature have been most extensively studied in conjunction with conventional antitumor therapies. Therefore, plant-based active substances can increase tumor cells’ sensitivity to classical antitumor therapy and reduce side effects associated with it [19]. Furthermore, the association between two or more phytochemicals has attracted the attention of researchers in the field, evidencing that the compounds may have an enhanced antitumor therapeutic effect [20,21].

Taking these premises into consideration, the aim of the present study was to evaluate the effects of digoxin (DG), betulinic acid (BA), and the combination of the two substances (DG + BA) on human melanoma cells—SK-Mel-28 and RPMI-7951—regarding the effect on the cell’s viability and morphology, as well as the structure of nuclei and actin filaments. Moreover, the irritation potential of the test samples was conducted by using the in ovo method on the chorioallantoic membrane of the hen’s egg.

## 2. Materials and Methods

### 2.1. Reagents

The present study employed the following reagents: (DG), (BA), dimethyl sulfoxide (DMSO), fetal calf serum (FCS), penicillin/streptomycin, alamarBlue (resazurin) and 4′,6-diamidino-2-phenylindole (DAPI) were purchased from Sigma Aldrich, Merck KgaA (Darmstadt, Germany). Rhodamine Phalloidin (00027) was procured from Biotium (Hayward, CA, USA). The culture medium specific to the cell lines, Eagle’s Minimum Essential Medium (EMEM—ATCC^®^ 30-2003TM), was acquired from ATCC (American Type Culture Collection, Manassas, VA, USA). All reagents used were approved for use on cell lines and were of analytical purity.

### 2.2. Cell Culture

In order to evaluate the impact of DG, BA, DG + BA, two human melanoma cell lines were selected—SK-Mel-28 (code number: HTB-72^TM^) and RPMI-7951 (code number: HTB-66^TM^), both obtained from the American Type Culture Collection (ATCC, LGC StandardsGmbH, Wesel, Germany). The cells were cultured in EMEM containing 10% FCS and 1% penicillin (100 U/mL)-streptomycin (100 μg/mL). Under standard incubator conditions, the cells were kept at 37 °C and 5% CO_2_ during the experiments.

### 2.3. Cellular Viability Assessment

The alamarBlue method was applied to test cell viability. After reaching a suitable confluence, of approximately 90%, the cells cultured in 96-well plates (1 × 10^4^ per well) were stimulated for a period of 24 h as follows: (i) betulinic acid (1, 5, 10, and 25 μM); (ii) digoxin (5, 10, 25, and 50 nM) and the combination between digoxin (5, 10, 25, and 50 nM) and betulinic acid (10 μM). Following the 24-h period, the culture medium was replaced with 200 μL of fresh culture medium and a volume of 20 μL of alamarBlue was added to each well, subsequently, the cells were incubated for 3 h at 37 °C. Finally, the absorbance was read at two wavelengths (570 and 600 nm) using Cytation 5 (BioTek Instruments Inc., Winooski, VT, USA).

### 2.4. Cellular Morphology

As part of the pharmaco-toxicological evaluation of the test samples, the microscopic evaluation of the cellular morphology was carried out to determine the potential impact at this level. Thus, the cells were observed and photographed under bright field illumination 24 h post-treatment with the concentrations previously tested in the cell viability assessment.

### 2.5. Immunofluorescence

To visualize the changes occurring at the level of the nucleus and actin fibers, SK-Mel-28 and RPMI cells were cultured in 12-well plates. Upon reaching 80–90% confluence, the cells were stimulated with BA 10 μM, DG 50 nM and the combination of the two DG 50 nM + BA 10 μM for 24 h. The cells were then washed three times with ice-cold PBS and fixed using 4% paraformaldehyde solution. Following fixation, the cells were permeabilized with 2% Triton X in phosphate-buffered saline (PBS). A blocking solution (30% FCS/0.01% Triton X-100) was added to block the effect of Triton X 2%. Rhodamine Phalloidin was added for 20 min to visualize the actin fibers, and 4,6-diamidino-2-phenylindole was added for 15 min to visualize the nuclei. Using an Olympus IX73 inverted microscope equipped with a DP74 camera, the images were captured and analyzed using CellSens V1.15 software (Olympus, Tokyo, Japan).

### 2.6. Chorioallantoic Membrane (CAM) Assay

An evaluation of the potential irritating effect on blood vessels was conducted using chicken eggs (Gallus gallus domesticus). The following steps were completed: (i) the eggs were washed and disinfected with 70% alcohol, after which the date was written and they were placed in the incubator; (ii) on the 4th day of incubation, a perforation was made in the tip of the eggs through which a volume of 6–7 mL of albumen was extracted in order to facilitate the detachment of the membrane from the inner shell of the hen’s egg; (iii) on the 5th day of incubation, a window was made in the upper part of the egg to visualize the blood vessels, after which the hole was covered with adhesive tape, and the eggs were placed in the incubator until the day of the start of the experiment.

### 2.7. Hen’s Egg Test—Chorioallantoic Membrane (HET-CAM) Assay

To evaluate the possible toxic and irritating potential of DG, BA, and their combination, on the level of blood vessels the HET-CAM method was applied. This experiment took place on the 10th day of incubation of the eggs, with the following steps: (i) the samples were added to the chorioallantoic membrane in a volume of 600 μL, so that the entire membrane was covered with liquid; (ii) a negative control (distilled water) and a positive control (sodium dodecyl sulfate, 1%) were used in parallel; (iii) the changes observed in the blood vessels were hemorrhage (H), vascular lysis (L) and coagulation (C), being noted the time when they were observed for the first time; (iv) the impact on the blood vessels was monitored for 5 min, obtaining photos both before applying the sample and after 5 min; (v) finally, the irritation score (IS) was calculated using a method previously described in the literature [22,23]:

$$IS=5\times \frac{301-H}{300}+7\times \frac{301-L}{300}+9\times \frac{301-C}{300}$$

A stereomicroscope (Discovery 8 Stereomicroscope from Zeiss, Göttingen, Germany) was used to visualize the changes occurring at the vascular level, and the photographs were taken with a Zeiss Axio CAM 105 color camera.

### 2.8. Combination Index Calculation

The combination of DG and BA was evaluated by applying the Chou and Talay principle, which consists of the calculation of the inhibitory effect (Fa), the dose reduction index (DRI), and the combined effect of the two compounds. An analysis of the synergistic effects was carried out using CompuSyn version 1.0 software (ComboSyn, Inc., Paramus, NJ, USA). By calculating the combination index (CI), the effect of the association of two compounds was determined, as follows: CI < 1 indicates a synergistic effect, CI = 1 indicates an additive effect, and CI > 1 indicates an antagonistic effect. Furthermore, a DRI value greater than 1 indicates that the dose of drugs used in combination can be reduced compared to monotherapy [24,25].

### 2.9. Statistical Analysis

The results are expressed as mean SD (standard deviation), and the difference between the groups was assessed using one-way ANOVA, followed by Dunnett’s multiple comparison post-test. The statistical analysis was performed using GraphPad Prism software version 9.0.0 for Windows (GraphPad Software, San Diego, CA, USA, www.graphpad.com). The statistically significant differences between data were labeled with * (**** *p* < 0.0001).

## 3. Results

### 3.1. Cellular Viability Assessment

In order to determine the cytotoxic potential of the two compounds, and their combination, the viability of human melanoma cells, SK-Mel-28 and RPMI-7951, was determined 24 h after stimulation with BA (1, 5, 10, and 25 µM), DG (5, 10, 25, and 50 nM) and DG (5, 10, 25 and 50 nM) + BA 10 µM.

**Figure 1:**
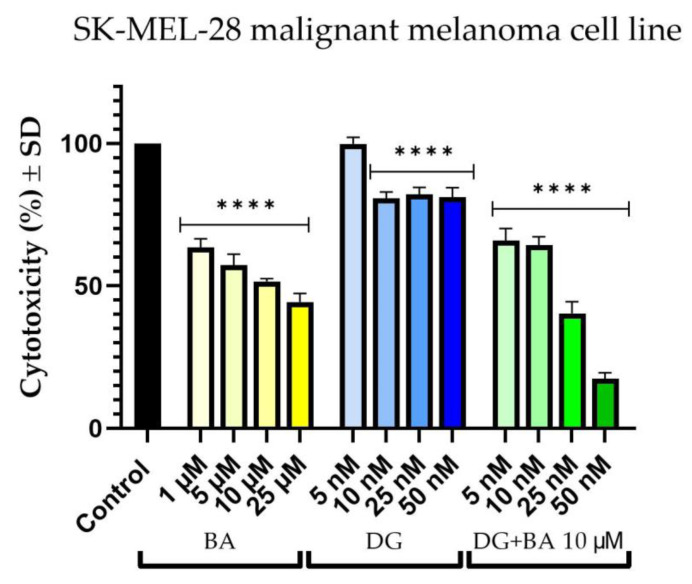
In vitro evaluation of the cytotoxic effects of BA (1, 5, 10, and 25 μM), DG (5, 10, 25, and 50 nM) and combination of DG (5, 10, 25, and 50 nM) + BA 10 μM on human melanoma cells—Sk-Mel-28 after 24 h of treatment. The results are expressed as percentages of viability (%) normalized to control cells (unstimulated cells) and expressed as the mean + standard deviation of three independent experiments performed in triplicate. Based on the one-way ANOVA analysis method, followed by Dunnett’s multiple comparison post-test, a statistical difference was determined between the tested and control cells (**** *p* < 0.0001).

On SK-Mel-28 cells, BA exhibits a concentration-dependent cytotoxic effect. Thus, at 1 μM, the percentage of viable cells of the melanoma cells decreased to approximately 63%. However, the most intense effect was observed at a concentration of 25 μM, which resulted in an approximately 44% decrease in cell viability. Furthermore, DG causes a slight reduction in cell viability. In this manner, at a concentration of 5 nM, cell viability is approximately 99%, while at concentrations of 10, 25, and 50 nM, the viability remains relatively constant at approximately 81%. As a result of combining DG + BA, the highest tested concentrations (25 and 50 nM) of DG accompanied by 10 μM of BA were observed to have a more pronounced cytotoxic effect than the two compounds tested individually. Thus, the strongest effect of inhibiting cell viability was recorded in the case of the combination of DG 50 nM and BA 10 μM, where cells’ viability percentage was approximately 17% (Figure 1).

**Figure 2:**
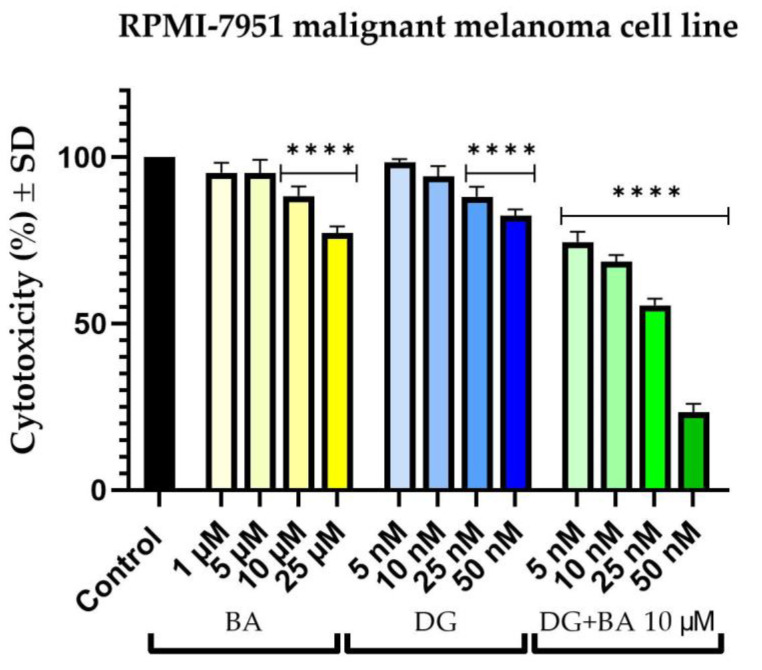
In vitro evaluation of the cytotoxic effects of BA (1, 5, 10, and 25 μM), DG (5, 10, 25, and 50 nM) and combination of DG (5, 10, 25, and 50 nM) + BA 10 μM on human melanoma cells—RPMI-7951 after 24 h of treatment. The results are expressed as percentages of viability (%) normalized to control cells (unstimulated cells) and expressed as the mean + standard deviation of three independent experiments performed in triplicate. Based on the one-way ANOVA analysis method, followed by Dunnett’s multiple comparison post-test, a statistical difference was determined between the tested and control cells (**** *p* < 0.0001).

A similar effect was also observed in RPMI-7951 cells. Accordingly, BA decreased the number of viable cells in a dose-dependent manner. Nevertheless, the effect of BA on cell viability was much less pronounced at the level of RPMI-7951 cells as compared with SK-Mel-28 cells. Therefore, in the case of the highest tested concentration, 25 μM, the most significant reduction in cell viability was observed, at approximately 77%. Similarly, DG reduced cells’ viability percentage in a concentration-dependent manner. As a result, at concentrations of 5 and 10 nM, cell viability was similar to the control cells, but at concentrations of 25 and 50 nM, viability decreased to approximately 88% and 82%, respectively. The combination, DG + BA, had a more pronounced cytotoxic effect than either compound alone. Thus, even at the lowest concentration tested, the percentage of viable cells decreased to a value of approximately 74%, while at the highest concentration tested, cells’ viability percentage dropped to a value of approximately 23% (Figure 2).

### 3.2. Cellular Morphology

To assess the cytotoxicity of the test samples, human melanoma cells were imaged and monitored after 24 h of treatment to determine if morphology changes had occurred.

**Figure 3:**
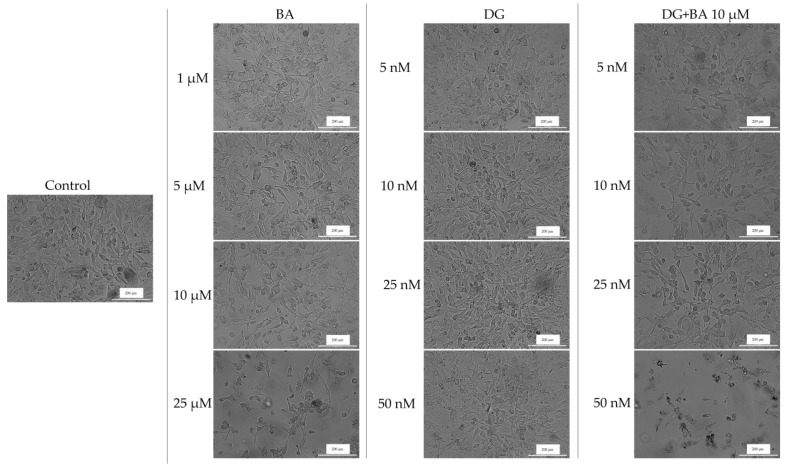
Morphological appearance and confluence of melanoma cells—SK-Mel-28 after 24 h of treatment with BA, DG, and DG + BA. Scale bars indicate 200 µm.

In human melanoma cells—SK-Mel-28, BA influenced cell confluence and morphology in a dose-dependent manner. At the lowest concentrations (1 and 5 μM), cell morphology does not change significantly, but cell confluency decreases slightly. Nevertheless, at a concentration of 25 μM, cells became round and detached from the plate, resulting in a marked decrease in cell confluency. Furthermore, in the case of DG, no significant changes in cell morphology were observed. The confluence and number of cells decreased slightly at a concentration of 50 nM; however, at 5, 10, and 25 nM, the cells have a similar morphology and confluence to those of the control cells. Conversely, the DG + BA has a more pronounced cytotoxic effect, especially at digoxin concentrations of 25 and 50 nM. The morphological changes observed in this case included rounding of the cells, detachment of the cells from the plate, and a decrease in cell confluency (Figure 3). These results are consistent with the results obtained in the case of the viability test.

**Figure 4:**
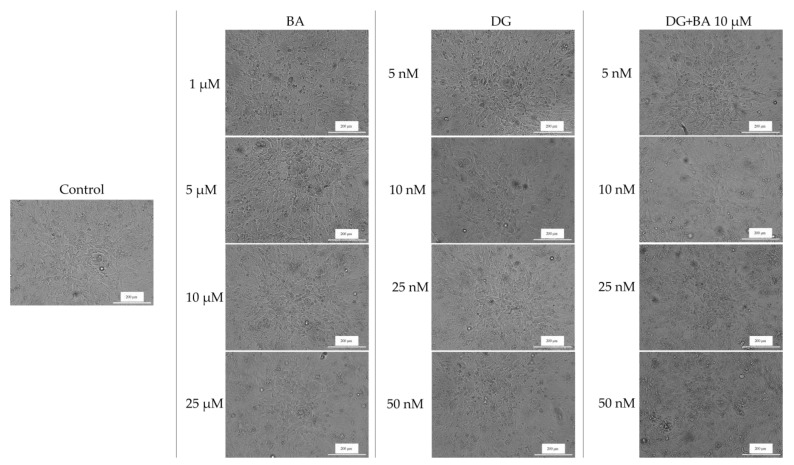
Morphological appearance and confluence of melanoma cells—RPMI-7951 after 24 h of treatment with BA, DG, and DG + BA. Scale bars indicate 200 µm.

The effects of BA on the cellular morphology of RPMI-7951 cells are relatively minor. Figure 4 shows that BA at concentrations of 1 and 5 μM does not significantly affect cell morphology and confluency, which are similar to those of control cells. Furthermore, at 25 μM, the shape of the cells changes slightly, becoming round and detached from the plate, but the number of affected cells is not as great as that of SK-Mel-28. The confluence and morphology of cells are also not significantly affected by DG. A rounded shape and a decrease in cell confluency were observed at the highest tested concentration, 50 nM, which was the most obvious effect. DG + BA, however, has a greater impact on cell morphology than the compounds alone. As a result, cell confluency decreased at 10 nM digoxin concentration. The 50 nM DG + 10 μM BA, however, led to a marked decrease in the number of viable cells as well as an intense change in cell shape, with numerous rounded cells being observed (Figure 4).

### 3.3. Immunofluorescence

The purpose of this assay was to investigate the changes occurring in the nucleus and actin fibers after stimulation with BA 10 μM, DG 50 nM, and the combination DG 50 nM + BA 10 μM to gain a deeper understanding of the mechanisms by which these compounds act on melanoma cells.

**Figure 5:**
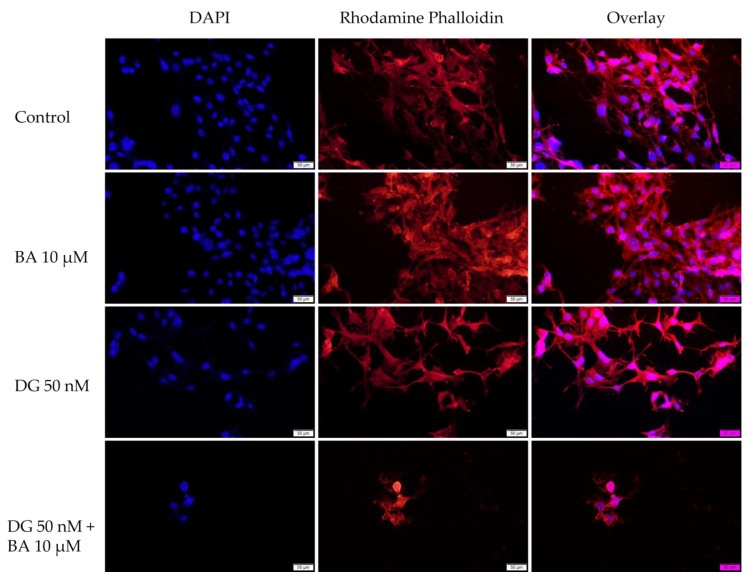
SK-Mel-28 cells visualized by fluorescence microscopy. The impact of BA 10 μM, DG 50 nM, and DG 50 nM + BA 10 at the level of: nuclei—DAPI staining (blue) and F-actin fibers—Rhodamine Phalloidin (red). The scale bar indicates 50 μm.

In SK-Mel-28 cells, BA, and DG cause only a slight condensation of chromatin and actin fibers. Actin fibers are found in condensed form around the nucleus in this case. As a result of the combination of DG 50 nM + BA 10 μM, the number of nuclei decreases and massive condensations of nuclei and actin fibers occur, indicating the onset of an apoptotic-like process (Figure 5).

**Figure 6:**
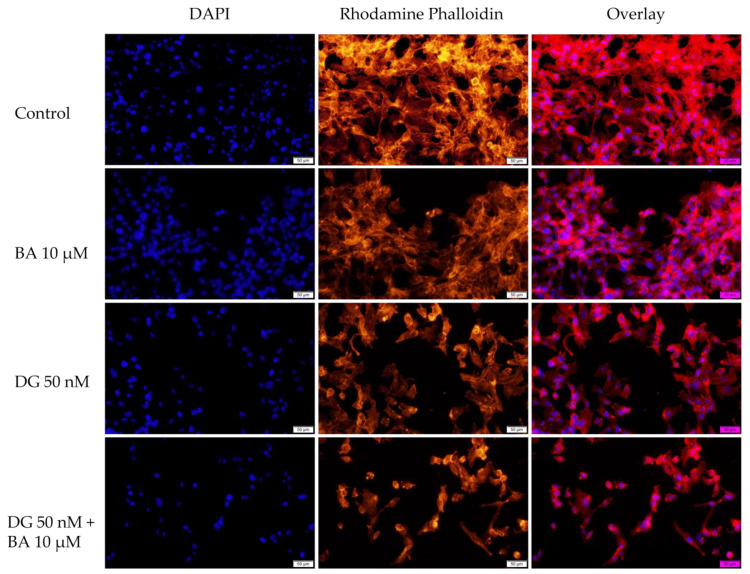
RPMI-7951 cells visualized by fluorescence microscopy. The impact of BA 10 μM, DG 50 nM, and DG 50 nM + BA 10 μM at the level of: nuclei—DAPI staining (blue) and F-actin fibers—Rhodamine Phalloidin (red). The scale bar indicates 50 μm.

Regarding the effect of BA on the nuclei and actin fibers of the RPMI-7951 cells, at a concentration of 10 μM causes a slight condensation of the nuclei and actin fibers. In the case of DG, a relatively low impact was observed on nuclei and actin fibers. However, the combination of DG + BA was found to have a strong effect on condensing nuclei and actin fibers, as well as causing the formation of apoptotic bodies (Figure 6).

### 3.4. Hen’s Egg Test—Chorioallantoic Membrane (HET-CAM) Assay

**Figure 7:**
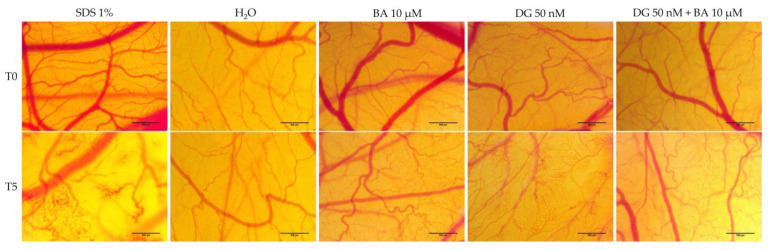
Stereomicroscopic images made before the application of the samples (T0) and five minutes after the application (T5) of CAMs inoculated with negative control—H_2_O (distilled water), positive control—SDS (sodium dodecylsulfate), BA 10 μM, DG 50 nM, and DG 50 nM + BA 10 μM (DG + BA).

The chorioallantoic membrane of the hen’s egg was used as a biological model for evaluating the toxic potential of BA (10 μM), DG (50 nM), and DG (50 nM) + BA (μM) at the level of blood vessels. A summary of the irritation scores obtained for the compounds, the positive control (SDS 1%) and the negative control (H_2_O) is presented in Table 1. Therefore, the highest irritation score was obtained in the case of the positive control, 19.86. Alternatively, the negative control, in which a score of 0.10 was obtained for irritation, stands at the opposite pole. In terms of the test samples, BA had an irritation score of 0.75, DG had a score of 1.09, and DG + BA had a score of 0.52 (Table 1). A slight intravascular coagulation, followed by a slight vascular lysis, was observed at the level of the vascular plexus following the administration of BA. However, DG showed a stronger irritating effect on the vascular system compared to BA. Vascular coagulation and vascular lysis were observed to be more pronounced in this case. The combination of DG + BA, however, did not result in significant changes, the only notable effect being a slight intravascular coagulation. Despite this, none of the compounds nor their combination showed significant irritation effects at the vascular level, and the viability of chicken embryos was not affected even after 24 h of application (Figure 7).

### 3.5. Combination Index Calculation

An assessment of the potential synergistic effect between digoxin and betulinic acid was conducted by applying the Chou–Talalay method. Digoxin and betulinic acid were combined in different ratios (2000:1, 1000:1, 400:1 and 200:1). With the assistance of CompuSyn software, the parameters were monitored and calculated.

Table 2 shows the inhibitory effect (Fa) values that were used to calculate the DRI and CI parameters for human melanoma cells—SK-Mel-28. At low concentrations of DG (5 and 10 nM), the CI values obtained indicate an antagonistic effect. In contrast, at higher concentrations (25 and 50 nM), the CI values (0.32 and 0.11) indicate an overall synergistic effect. DRI values of 25 and 50 nM of digoxin were greater than 1, indicating a favorable reduction in the concentration of DG and BA combined (Table 2).

For the human melanoma cell line RPMI-7951, the inhibitory effect (Fa) values used to calculate the DRI and CI parameters were: 0.74; 0.68; 0.55, and 0.23. The CI values were all below 1, indicating a strong synergistic effect. As well, DRI values were greater than 1 for all tested DG concentrations, suggesting the possibility of reducing the doses of DG or BA when used together (Table 3).

## 4. Discussion

Digoxin is one of the oldest drugs used in medical practice, particularly in the treatment of cardiovascular disease. Following the observation that patients treated with digoxin developed mostly benign tumors, the first hypothesis about digoxin’s antitumor activity was formed [26]. To date, several studies have demonstrated that digoxin has an antitumor effect on several types of cancer [27,28,29]. As far as the antitumor effect in melanoma is concerned, digoxin has been proven to be effective in combination with conventional antitumor treatments [16]. Additionally, betulinic acid, as a member of the pentacyclic triterpene class, is well known for its range of biological properties, including its antitumor potential. Several studies have proven the effectiveness of betulinic acid in affecting melanoma cells through various mechanisms such as the induction of apoptosis and alteration of cellular respiration [9,30,31]. The simultaneous administration of several antitumor drugs with different therapeutic targets has been shown to improve therapeutic efficacy. Furthermore, the plant kingdom has allowed the discovery of new natural alternatives that have drawn significant attention in recent years as a result of their low toxicity as compared to traditional chemotherapy. Consequently, the literature provides evidence that the combination of antitumor agents with different natural compounds is effective [32]. Based on these premises, the present study proposed to evaluate in vitro and in ovo the effectiveness of combining two compounds, betulinic acid and digoxin, against human melanoma cells.

The first objective of this study was to evaluate in vitro the effects of BA (1, 5, 10, 25, and 50 μM) and DG (5, 10, 25, and 50 nM) along with the combination of all four concentrations of DG and BA at a concentration of 10 μM. It was decided to investigate the antitumor potential of these compounds on two human melanoma cell lines—SK-Mel-28 and RPMI-7951—by measuring cell viability, cell morphology, and changes in nuclei and actin fibers. Based on the results of the cell viability experiments, both compounds, as well as the combination between them, decreased cells’ viability percentage in both cell lines. There was, however, a greater cytotoxic effect observed when DG 50 nM was combined with BA 10 μM, leading to a significant decrease in cell viability (SK-Mel-28 cells—17%, and RPMI-7951 cells—23%). Cell viability was determined using the alamarBlue assay. Over the past 50 years, alamarBlue has been used to determine cell viability, and is one of the most frequently used methods [33]. Several advantages of this method were considered in the current study, including its low toxicity and reduced interference with cellular metabolism. In addition, Hamid et al. demonstrated in their study that alamarBlue has a much higher sensitivity compared to other methods of measuring cell viability, and it does not interfere with electron transport [34]. The selection of concentrations of betulinic acid and digoxin for the present study was based on an extensive review of the literature in this field, as presented below.

Recent studies have focused on betulinic acid in light of its many therapeutic properties. As far as antitumor activity is concerned, betulinic acid has been found effective in a number of cancer types, including prostate carcinoma, breast carcinoma, and colorectal carcinoma [35,36,37]. According to several studies, BA poses a high level of cytotoxicity for melanoma cells. A pioneering study on the effects of betulinic acid on melanoma cells was conducted by Pisha and colleagues [10]. It was later indicated by Tan et al. that BA phosphorylates pro-apoptotic proteins in melanoma cells. In this case, betulinic acid was tested at 4 and 8 μg/mL, corresponding to 8.75 and 17.5 μM [30]. Similarly, Pfarr and colleagues evaluated the effect of BA at concentrations between 1 and 150 μM on the melanoma cell line—B164A5. Based on the results of the study, the concentration of 15 μM determined a viability of less than 20% in melanoma cells, which is indicative of the strong cytotoxic effect of BA at low concentrations [38]. BA was also tested on melanoma cells—A375, and it was concluded that a concentration of 10 μM interfered with the epithelial-to-mesenchymal transition, whereas a concentration of 50 μM significantly reduced cell proliferation [39]. Furthermore, it has been shown that BA in subtoxic concentrations (10 μM) induces mitochondrial dysfunction in A375 melanoma cells. BA also showed a concentration-dependent cytotoxic effect that induced signs of apoptosis at concentrations between 1 and 50 μM [40]. A key advantage of betulinic acid is its low toxicity at the level of healthy cells. Researchers have shown that normal cells of different origin are more resistant to BA’s action than cancerous cells, suggesting that BA is selectively toxic to tumor cells [41,42,43,44]. In addition to betulinic acid therapy, it may also be used in conjunction with conventional antitumor therapies. Selzer et al. evaluated the effects of BA alone and in combination with ionizing radiation on melanoma cell lines MES20, MES21, A375, and 518A2. Using BA in conjunction with ionizing radiation strongly suppressed tumor cell proliferation. In addition, BA did not elicit any strong cytotoxic effects on healthy melanocytes. Therefore, in combination with ionizing radiation, BA exerts additive effects, resulting in greater and more selective effectiveness against melanoma [41]. Additionally, BA was evaluated in combination with paclitaxel and docetaxel on melanoma cells. According to the study, BA has no cytotoxic effects on human keratinocytes—HaCaT, while BA, on the other hand, displayed cytotoxic effects on tumor cells, exhibiting an IC50 value between 2.21 and 15.94 μM. It was also found that additive effects could be obtained through the association with paclitaxel and docetaxel [45].

The possibility of using digoxin as a treatment for a wide variety of cancers has been suggested by epidemiological studies [26,46]. A study conducted by Wang et al. evaluated the effects of digoxin at concentrations between 25 and 100 nM on two lung cancer cell lines. It was noted that digoxin exhibits a concentration-dependent cytotoxic effect and, in addition, when combined with Adriamycin, it produces a synergistic effect on tumor cells [27]. Similarly, Chou and colleagues studied the effect of DG in concentrations between 10 and 100 nM on the SKOV-3 ovarian cancer cell line. There was also a concentration-dependent cytotoxic effect in this case, with the lowest value of cell viability obtained at a concentration of 100 nM of digoxin [47]. In a recent clinical trial, DG was evaluated for the treatment of melanoma. A DG dose of 0.25 mg per day was administered in combination with trametinib 2 mg. According to the results of the study, melanoma patients showed an improvement in their response to treatment [16]. Being used for more than six decades in therapy, digoxin has a well-established safety profile. For patients suffering from heart failure or atrial fibrillation, digoxin is usually prescribed at a dose of 0.25 mg daily. The onset of digoxin toxicity usually begins at concentrations of 1.2 ng/mL or even higher [48]. In the present study, the doses used in vitro and in vivo were correlated based on the calculation formula described by Levy [49]. Since the distribution volume of digoxin in healthy individuals is 7 L/kg, and the toxic concentration is considered 1.2 ng/mL, the correlated in vitro concentration is 365 nM. Therefore, the concentrations tested in the present study were chosen in order to be much lower than toxic doses.

Based on the morphology and the impact on nuclei and actin fibers, the combination DG 50 nM + BA 10 μM showed the greatest impact. There were a number of phenomena observed in this case, including the round shape of the cells, their separation from the plate and the decrease in cell confluence, and on the other hand, strong condensed nuclei and actin fibers and the appearance of apoptosis bodies. Over the past 150 years, changes in cell morphology have played an important role in describing the type of cell death that occurs [50]. Further, recent advances in molecular biology have contributed significantly to the increasing understanding of cell morphology. The process of cells being detached from the plaque is characteristic of apoptosis of monolayer adherent cells and is known as anoikis [51]. At the beginning of the apoptosis process, cells lose contact with their neighboring cells. Afterwards, the cells exhibit protrusions at the plasma membrane level, called blebs. Eventually, the cells shrink, and the blebs separate to form apoptotic bodies. Thus, apoptosis is characterized by the contraction of cells, the formation of blebs, and the formation of apoptotic bodies [52]. A similar pattern of changes was observed among melanoma cells treated with BA, DG, and, in particular, BA + DG.

Cell morphology can provide insight into the type of cell death involved. Thus, when apoptosis occurs, organelles and membranes are not directly affected, instead, the nucleus suffers early degeneration. Necrotic cells, on the other hand, have a relatively unaffected nucleus, whereas early degeneration occurs at the level of the cell membrane and organelles [53]. During the process of cell apoptosis, chromatin can undergo a transition from a heterogenous network, which is genetically active, to a compact, condensed, and inert form, which is then fragmented and packed into apoptotic bodies [54]. The actin protein is one of the most abundant proteins in cells. Actin cytoskeletons play a crucial role in many cellular functions, including apoptosis. The spatial dynamics of the actin cytoskeleton undergo rapid changes in response to the changes occurring at the cellular level. In the course of apoptosis, caspases are activated, which cause changes at the cellular level, including chromatin condensation, contraction of cells, nuclear fragmentation, and the formation of apoptotic bodies. Additionally, the actin cytoskeleton undergoes reorganization and condensation during apoptosis, which results in the formation of a peripheral cortical actomyosin ring [55]. As noted above, all these morphological and structural changes were observed in the present study, in melanoma cells treated with DG + BA, which suggests that this combination may trigger apoptosis in tumor cells.

Using the hen’s egg chorioallatoic membrane as a biological model, the present study was also intended to evaluate the toxic potential at the level of the vascular plexus. Based on the results obtained, BA 10 μM had an irritation score of 0.75, DG 50 nM had an irritation score of 1.09, and DG 50 nM + BA 10 μM had an irritation score of 0.52. As a result of the calculated irritation score, the substances can be divided into: (i) non-irritants (IS = 0–0.9), (ii) irritating substances (IS = 1–8.9), and (iii) strongly irritating substances (IS = 9–21). It is worth noting that the chorioallantoic membrane of the chicken embryo is a complete tissue that includes capillaries, arteries, and veins. Taking advantage of this approach, the toxic potential of different substances can be studied easily, as they are placed in direct contact with the membrane, and the potential inflammatory effect at the level of the conjunctival blood vessels can be examined [56]. Accordingly, BA 10 μM and the combination of DG 50 nM + BA 10 μM had no irritating effects at the vascular level, whereas DG 50 nM had a slight irritating effect, having the highest irritation score of 1.09. However, none of the compounds tested had significant toxic effects; even 24 h after the application of the samples, the viability of the embryos was not affected. Betulinic acid has been studied previously at the level of hen’s egg chorioallantoic membranes for antiangiogenic effects. Therefore, Qi and colleagues examined the antiangiogenic effect of BA incorporated in a mycelial system composed of grafted copolymers at the chorioallantoic membrane level. Their findings indicate that BA has a strong antiangiogenic effect [57]. According to Dehelean et al., who evaluated the effects of betulinic acid in the form of a nanoemulsion at the level of the chorioallantoic membrane, a strong antiangiogenic effects were also observed [58]. Further, Coricovac et al. demonstrated that BA inhibited both angiogenesis at the level of the chorioallantoic membrane and the development of melanoma tumors in ovo [59]. Conversely, Rodrigues and colleagues assessed the effect of digoxin delivered through poly(ε-caprolactone) implants. Following 5 min of exposure at the chorioallantoic membrane level, digoxin did not affect the vascular plexus. In contrast, after 24 h of exposure to digoxin at a dose of 2.88 μg, the chorioallantoic membrane shows signs of hemorrhage and intravascular coagulation [60]. Moreover, Svensson et al. evaluated digoxin’s effects at the level of CAM, showing that it inhibits angiogenesis [61]. Accordingly, this study is based on literature data and is supplemented with new innovative elements, such as a systematic in vitro analysis of digoxin along with betulinic acid at the level of human melanoma cell lines—SK-Mel-28 and RPMI-7951—in terms of cell viability, cell morphology, and influence on nuclei and actin fiber structure. Moreover, as far as we know, the combination of DG and BA has not been evaluated in terms of its irritating effect on the chorioallatoic membrane. In sum, all these data provide a preliminary assessment of the possible synergistic antimelanoma effects of digoxin and betulinic acid.

## 5. Conclusions

A key objective of the present study was to assess the antitumor potential of betulinic acid, digoxin, and their association at the cell level of two human melanoma cell lines, as well as the irritant potential of the chorioallantoic membrane of hen eggs, in ovo. The data from this study revealed that a DG 50 nM +BA 10 μM combination presented a more pronounced cytotoxic effect than that of the compounds tested individually at the level of both types of tumor cells, resulting in a decrease in cell viability and morphological changes, as well as changes at the level of the nuclei and actin fibers, indicating an apoptotic-like effect. Aside from this, there were no toxic effects observed in ovo on blood vessels. These data provide a basis for future pharmaco-toxicological studies to deepen the synergistic effect exerted on melanoma cells and to establish the biological mechanisms involved.

## Figures and Tables

**Table 1:** The irritation score obtained after applying to the chorioallantoic membrane the negative control (H_2_O), the positive control (SDS 1%), BA (10 μM), DG (50 nM), and DG 50 nM + BA 10 μM.

	H_2_O	SDS 1%	BA 10 μM	DG 50 nM	DG 50 nM + BA 10 μM
IS	0.10	19.86	0.75	1.09	0.52
tH	300	20 s	300	296	300
tL	300	18 s	290	285	295
tC	299	15 s	285	280	289

IS—irritation score; tH—hemorrhage time; tL—lysis time; tC—coagulation time; H_2_O—distilled water; SDS—sodium dodecylsulfate.

**Table 2:** Inhibitory effect (Fa), combination index (CI) and dose reduction index (DRI), for betulinic acid and digoxin combination in SK-Mel-28 cells.

Inhibitori Effect (Fa)	Combination Index (CI)	Dose BA	Dose DG	Dose Reduction Index (DRI) BA	Dose Reduction Index (DRI) DG
0.65	11.9064	10 μM	5 nM	0.08454	12.7600
0.64	10.0020	10 μM	10 nM	0.10151	6.62483
0.4	0.32406	10 μM	25 nM	6.17001	6.17336
0.17	0.11826	10 μM	50 nM	864.114	8.53929

**Table 3:** Inhibitory effect (Fa), combination index (CI) and dose reduction index (DRI), for betulinic acid and digoxin combination in RPMI-7951 cells.

Inhibitori Effect (Fa)	Combination Index (CI)	Dose BA	Dose DG	Dose Reduction Index (DRI) BA	Dose Reduction Index (DRI) DG
0.74	0.24457	10 μM	5 nM	5.77905	13.9796
0.68	0.20759	10 μM	10 nM	9.93477	9.35133
0.55	0.19019	10 μM	25 nM	27.7052	6.48955
0.23	0.07838	10 μM	50 nM	377.738	13.2049

## Data Availability

Not applicable.
